# Effect of bispectral index-guided total intravenous anesthesia in younger children: A prospective, randomized, controlled trial

**DOI:** 10.3389/fneur.2022.1028582

**Published:** 2022-11-10

**Authors:** Guoliang Liu, Jianmin Zhang, Fang Wang, Lijing Li, Xuemei Zhang

**Affiliations:** Department of Anesthesiology, National Center for Children's Health, Beijing Children's Hospital, Capital Medical University, Beijing, China

**Keywords:** bispectral index, younger children, total intravenous anesthesia, recovery, propfol

## Abstract

**Background:**

BIS-guided total intravenous anesthesia (TIVA) is widely used in children, but few studies have attempted to evaluation of the effect of BIS-guided TIVA in younger children. This study aimed to evaluate the effect of bispectral index (BIS) guidance during TIVA in younger children during anesthesia.

**Methods:**

This study is a prospective, randomized, single-blind and controlled clinical trial. This study enrolled pediatric patients (aged 1–3 years) who were scheduled for surgery under TIVA with propofol and remifentanil. The children were randomly assigned to the BIS group (group B) and standard clinical practice group (group S). The BIS values in group B were maintained at 45–60. The anesthesiologist controlled the depth of anesthesia in group S according to the variation in the clinical signs of the children. The time of extubation, duration of stay in post-anesthesia care unit (PACU), as well as BIS values, heart rate (HR), mean arterial pressure (MAP), pulse oxygen saturation at eight time points 1 min before induction (T1), 1 min after induction (1 min after the induction drugs were administrated) (T2), immediately after intubation (T3), immediately after skin incision (T4), 30 min after the start of the operation (T5), 60 min after the start of operation (T6), immediately after drug withdrawal (T7), and immediately after extubation (T8), propofol consumption, and postoperative adverse reactions were recorded.

**Results:**

There was no significant difference in time to extubation 15(10,21) vs 14 (11,20) and duration of stay in PACU 27 (20,37) vs. 29 (22,39) between the group B and group S. At the time points 30 min after the start of the operation, 60 min after the start of operation and immediately after drug withdrawal, the BIS values in group S were significantly higher than those in group B (57 ± 9, 57 ± 9, 60 ± 8 vs 52 ± 7, 54 ± 7, 57 ± 6).

**Conclusions:**

The use of BIS-guided total intravenous anesthesia in younger children does not shorten the time of extubation and the duration of stay in the PACU.

**Trial registration:**

Chictr.org.cn identifier: 24/11/2017, ChiCTR-IOR-17013530.

## Background

Compared to inhalation anesthesia, total intravenous anesthesia may present clinical advantages. Recent studies have demonstrated that propofol anesthesia in children, as in adults, is associated with a major reduction in post-anesthetic nausea and vomiting incidence (1), with a decrease in emergence agitation episodes (2) and a better quality of recovery (3). In addition, TIVA is widely accepted by anesthesiologists because it reduces passive inhalation by anesthesiologists. Currently, the latest application method of TIVA involves using a target-controlled infusion pump to induce and maintain anesthesia. However, the target-controlled infusion pump is not available in all countries and hospitals; therefore, a manual infusion regimen based on varying infusion rates is widely used in children (4). The dose and speed of manual infusion are judged and adjusted by pediatric anesthesiologists according to the clinical parameters of the children. Because many factors interfere with clinical parameters, it is difficult to adjust the anesthesia depth objectively, quantitatively, and accurately (5). Therefore, continuous propofol infusion under the guidance of BIS or other monitoring indexes has gradually gained application in pediatric anesthesia. Although the algorithm of BIS is calculated based on adult research, after years of research, there have been many literature studies that BIS are safe and feasible in children's sedation and anesthesia, and have a good role in guiding the depth of anesthesia (6–14). Previous studies have shown that propofol combined with remifentanil infusion under auditory-evoked potential monitoring can reduce propofol consumption and time to the emergency (15). Still, studies show that BIS-guided propofol infusion has no advantage over conventional anesthesia in propofol consumption and emergency times (16). Regardless of the results of previous studies, there are still fewer studies on the effect of BIS-guided TIVA than that on conventional methods in younger children.

The objective of this prospective, randomized, controlled study was to evaluate the effects of BIS monitoring TIVA in younger children on time to extubation and on the quantity of anesthetics compared with the conventional anesthesia method.

## Methods

### Ethical approval and consent to participate

This study was approved by The Ethical Committee of Beijing Children's Hospital, Capital Medical University, National Center for Children's Health with approval number 2016–99. The children‘s parents provided written consent. All the experiment protocol for involving human date and methods were in accordance with the guidelines of Declaration of Helsinki.

### Study population

From December 2017 to June 2019, children undergoing elective operations, such as urologic, orthopedic, and surgical oncology procedures, under general anesthesia were consecutively enrolled. The inclusion criteria were as follows: children who received endotracheal intubation under TIVA, whose ages were between 1 and 3 years, and whose ASA scores were I to II. The exclusion criteria were as follows: children with intraoperative use of inhaled anesthetics, those who underwent cardiopulmonary bypass surgeries for congenital heart disease, craniocerebral surgeries, where BIS electrodes could not be placed, and surgeries with an estimated operative time < 60 min. Children with a history of nervous system disease, use of related psychoactive medications, and a history of allergy to the study drug were likewise excluded. Children were randomized into the BIS group (group B) and the standard clinical practice group (group S) using SAS 9.4 software, with 215 children in each group. The anesthesia depth of children in group B was accurately adjusted according to the BIS value. The anesthesia depth of children in group S was adjusted according to the judgment of the clinical parameters by the anesthesiologist.

### Anesthesia method

None of the patients were pre-medicated. Children were routinely fasting from food for ≥6 h and from drinking for ≥4 h before surgery. Peripheral intravenous access was established in all patients in the hospital ward. A continuous monitor (MP70 monitor; Philips Intellivue, Germany) was used for noninvasive blood pressure, pulse oxygen saturation, heart rate (HR), and electrocardiogram monitoring in the operating room. BIS electrodes were attached to the forehead of the child, and a BIS monitoring instrument (Aspect Medical System, USA, Version 3.0) was connected and continuously monitored.

All children underwent induction with 2–3 mg/kg of propofol (1% propofol in a medium-chain/long-chain triglyceride emulsion; Fresenius Kabi Deutschland Gmbh, Germany), 0.2–0.3 μg/kg of sufentanil (Yichang Humanwell Pharmaceutical Co., Ltd., Hubei Province, China) or 2–3 μg/kg of fentanyl (Yichang Humanwell Pharmaceutical Co., Ltd., Hubei Province, China), 0.5–0.7 mg/kg of rocuronium (Hameln Pharmaceuticals Gmbh, Germany), followed by endotracheal intubation after a sufficient depth of anesthesia was achieved. After intubation, the anesthesia machine (Datex-Ohmeda Inc., Madison, USA) was connected to maintain mechanical ventilation, with a tidal volume of 8–10 ml/kg, a frequency of 15–20 beats/min, and *I*: *E* = 1:2, End-tidal carbon dioxide partial pressure 35–45 mmHg. An intravenous injection pump (Smiths Medical Instrument Co., Ltd., Zhejiang Province, China) was then connected for continuous intravenous infusion of propofol 4–12 mg/kg/h and remifentanil (Yichang Humanwell Pharmaceutical Co., Ltd., Hubei Province, China) 0.2–0.4 μg/kg/min to maintain anesthesia. For pediatric patients in group B, the anesthesiologist adjusted the infusion rate of propofol according to the BIS values and tried to keep the BIS values between 45 and 60. Pediatric patients in group S were also monitored for BIS, but the anesthesiologist was blinded to the BIS value, and the research nurse recorded the BIS value. The anesthesiologist controlled the anesthesia depth according to routine methods of HR, BP, and surgical simulation. After skin closure, all anesthetic infusions were discontinued, and the endotracheal tube could be removed when the child's protective reflexes such as choking and swallowing were restored. There was an eye-opening reflex by patting the cheek. The patients were sent to the post-anesthesia care unit (PACU) and continued to be observed and evaluated by the nurses who were unaware of the study design and grouping in the recovery room. The patients could return to the ward only after the total score of the three items was above 4 points (*A* + *B* + *C* ≥ 4 points). The Steward Awakening Score Scale (17) is presented in Table 1.

**Table 1:** Steward awakening score scale.

		**2**	**1**	**0**
A	Level of consciousness	Full recovery	Responsiveness to stimulation	No responsiveness to stimulation
B	Degree of the unobstructed respiratory tract	cough on request	Maintenance of respiratory tract unobstructed without support	Requirement for respiratory support
C	Physical activity	Moving of limbs purposefully	Non-purposeful moving of limbs	No motoric activity of limbs

### Data collection

The primary endpoint was the time of extubation (the time from stopping all anesthetic infusions to the removal of the endotracheal tube). The secondary outcomes of interest were the duration of stay in the PACU (the time from being transferred to the PACU after extubation to meet the criteria for leaving the recovery room), the total amount of propofol and the BIS values, heart rate, mean aterial pressure, and pulse blood oxygen saturation values at 1 min before induction (T1), 1 min after induction (1 min after the induction drugs were administrated) (T2), immediately after intubation (T3), immediately after skin incision (T4), 30 min after the start of the operation (T5), 60 min after the start of operation (T6), immediately after drug withdrawal (T7), and immediately after extubation (T8). The incidence of anesthesia-related adverse reactions and adverse events was observed and followed up.

### Randomization

Stratified randomization with anesthetist as stratum will be performed. The randomization number will be generated by the statistical professionals using SAS 9.4. Each subject who qualifies for entry into the study will be assigned a patient number according to the time order. Each anesthetist will be provided with a randomization list (using blocks). Then, a patient scheduled for surgery would be first assigned to an anesthetist and then randomized to the study group. Each number corresponds to the randomization and allocates the subject to one of the groups (1:1 group allocation).

### Blind method

This study is a single-blind trial. The subjects of both groups will accept continuous BIS monitoring, and will not know whether they belong to the study group or the control group before or after anesthesia. During the anesthesia process, the anesthesiologist conduct the anesthesia guided by BIS and that guided by standard practice respectively according to random grouping results. In group S were also monitored for BIS, but the anesthesiologist was blinded to the BIS value, and the research nurse who was not involved in the anesthesia recorded the BIS value.

### Statistical analysis

The sample size was calculated based on previous similar literature results (18), with the time of extubation being the primary outcome measure. The case ratio of the experimental group to the control group was 1:1, and the independent sample *t*-test method was used in the case of α = 0.025 (two-sided), power (1–β) = 90%. The calculation result of the sample size was 165 children in each group, and the estimated dropout rate was 30%. Therefore, the sample size of this study was approximately 215 per group, with a total of 430 children.

IBM SPSS (version 21.0; SPSS Inc., Chicago, IL, USA) was used for data analysis. Normally distributed numerical data are expressed as mean ± standard deviation (SD; *x* ± *s*). The measurement data of skewed distribution were expressed as the median (interquartile range-IQR). Repeated measures ANOVA was used to analyze BIS values, MAP, HR at multiple time points in two groups. A *post-hoc* Bonferroni test was used to compare differences at same time points in two groups and at different time points in same groups. The nonparametric test was used to analyze the data of skewed distribution. Statistical significance was set at *p* < 0.05.

## Results

**Figure 1:**
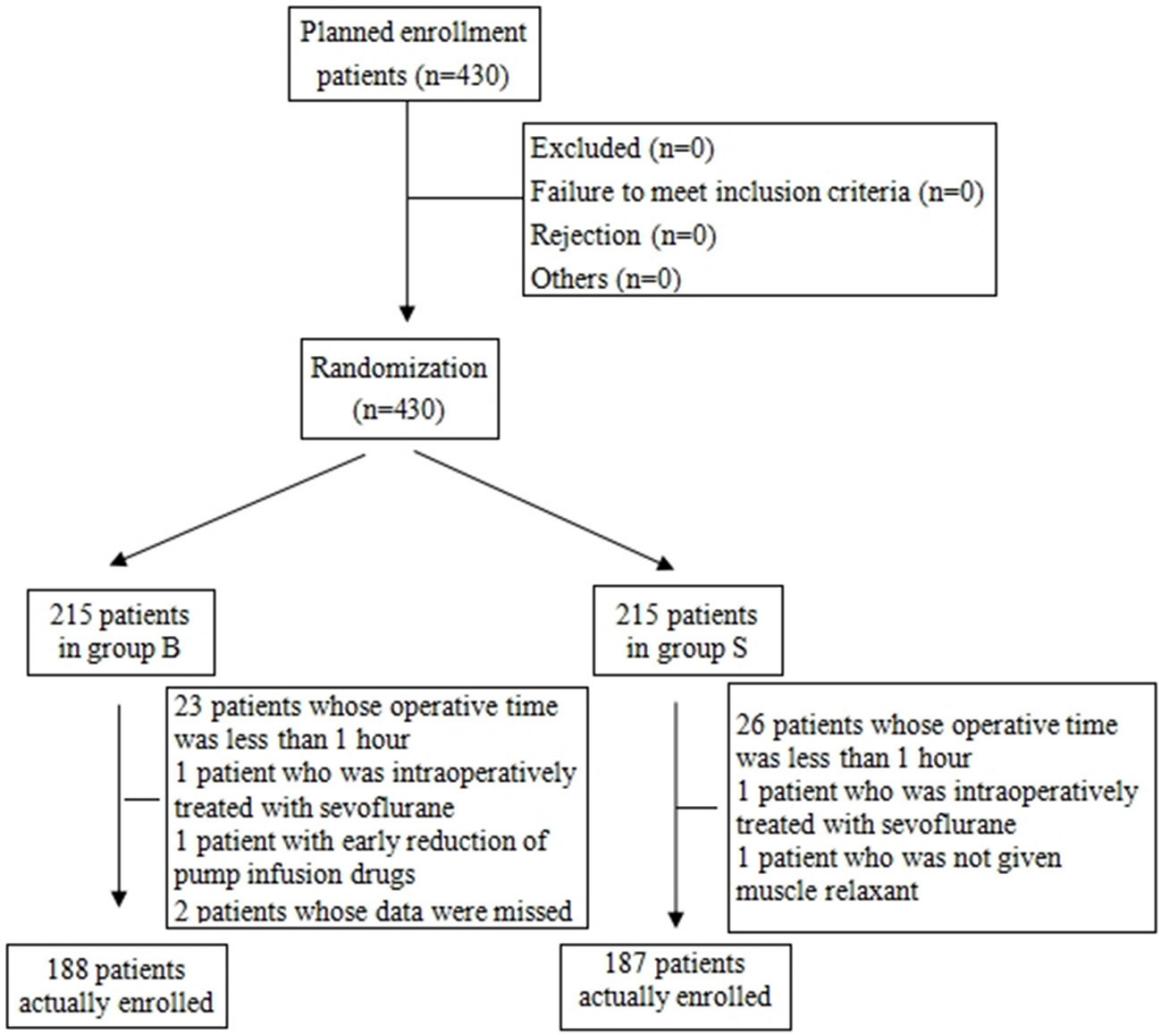
Flowchart showing the process of enrollment of all children.

**Figure 2:**
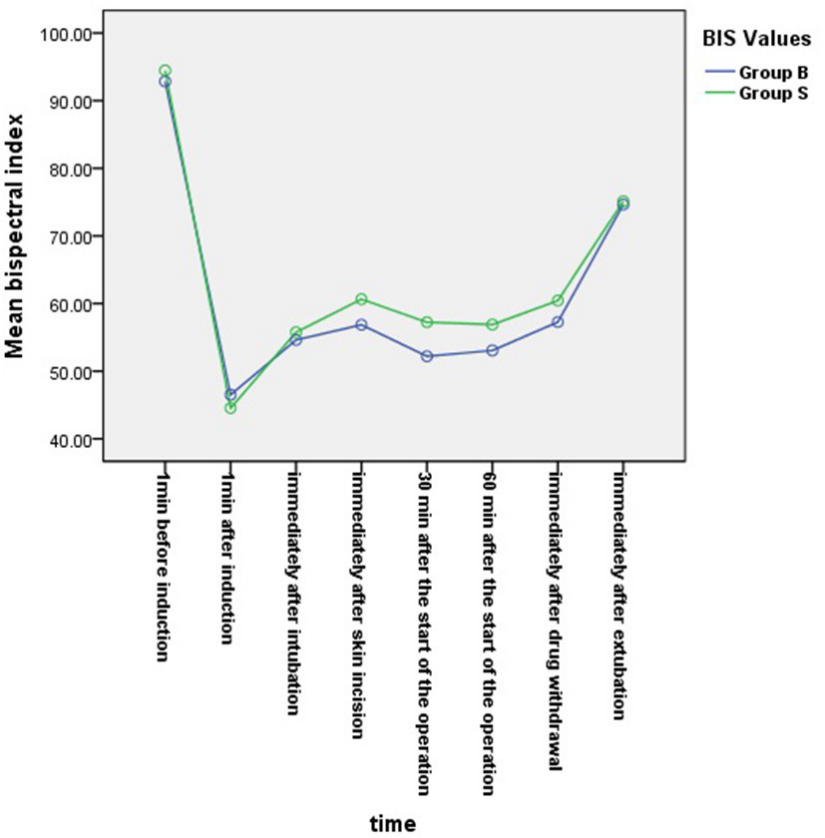
Changes in bispectral index (BIS) values at each time point in the two groups. BIS values significantly differed among the eight time points as well as between the two groups (*P* < 0.05). At the time points 30 min after the start of the operation, 60 min after the start of operation and immediately after drug withdrawal, the BIS values in group S were significantly higher than those in group B (*P* < 0.05).

**Figure 3:**
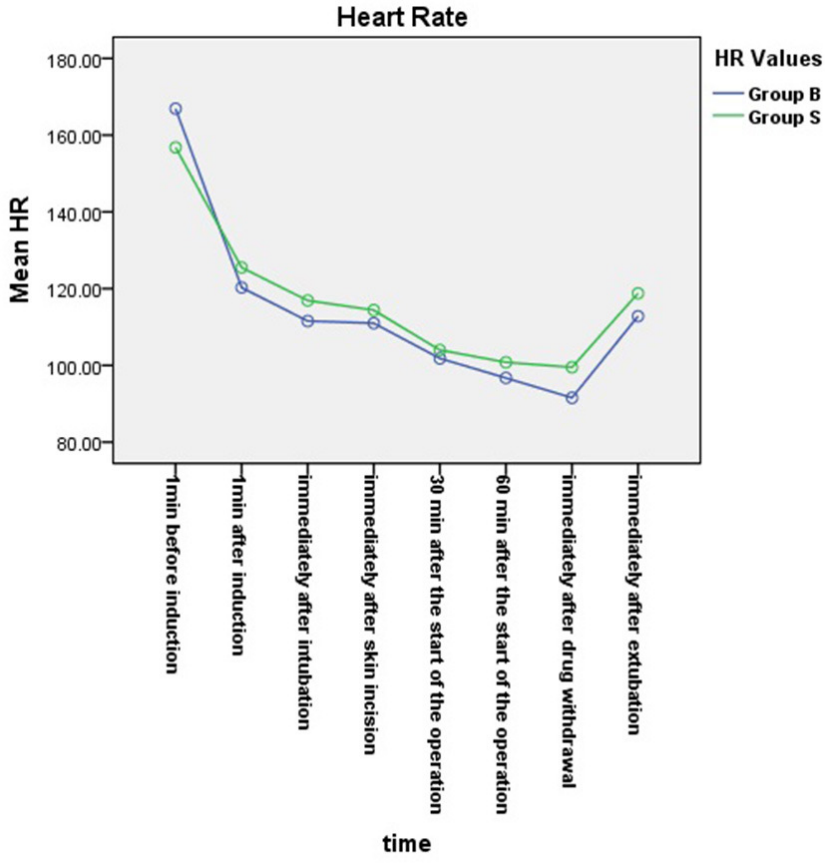
Changes in heart rates at each time point in the two groups. The HR values significantly differed among the eight time points (*P* < 0.05). There was no significantly difference in HR values between the two groups (*P* > 0.05).

**Figure 4:**
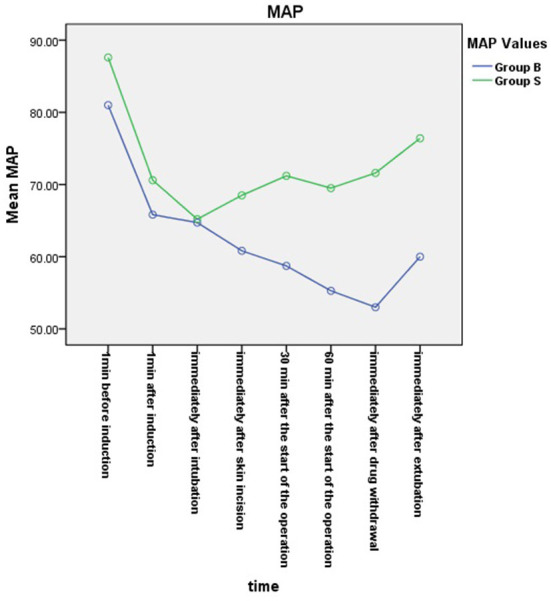
Mean arterial pressure (MAP) changes in the two groups. The MAP values significantly differed among the eight time points as well as between the two groups (*P* < 0.05). MAP in group B was lower than in group S at 30 min after the start of the operation, 60 min after the start of operation, immediately after drug withdrawal, and immediately after extubation (*P* < 0.05).

From the 430 initially recruited patients, 55 were excluded. Twenty-seven patients in group B were excluded, including 23 patients whose operative time was less than 1 h, one patient who was intraoperatively treated with sevoflurane, one patient with early reduction of infusion pump drugs, and two patients whose excessively long operative time imposes serious bias on the statistical data. Thirty-eight patients in group S were excluded, including 26 patients whose operative time was less than 1 h, one patient who was intraoperatively treated with sevoflurane, and one patient whose muscle relaxant was not administered at induction. A total of 375 children were included in the analyses: 188 in group B and 187 in group S (Figure 1). General information of the two groups of children is shown in Table 2, and there was no statistical difference in sex, age, weight, and operation type (*P* > 0.05). There was no significant difference in extubation time 15 (10,21) vs. 14 (11,20), duration of stay in the PACU time 27 (20,37) vs. 29 (22,39) and anesthesia time 111 (83,141) vs. 111 (82,144) between the two groups (*P* > 0.05). There was a significant difference in the total amount of propofol consumed between the two groups (*P* < 0.05), and group B consumed more propofol than group S (Table 3). The BIS values, HR and MAP of two groups were recorded in Table 4. Repeated measures ANOVA showed that BIS values significantly differed among the eight time points as well as between the two groups (*P* < 0.05). At the time points 30 min after the start of the operation, 60 min after the start of operation and immediately after drug withdrawal the BIS values in group S were significantly higher than those in group B (*P* < 0.05), as shown in Figure 2. The HR values significantly differed among the eight time points (*P* < 0.05). There was no significantly difference in HR values between the two groups (*P* > 0.05), as shown in Figure 3. The MAP values significantly differed among the eight time points as well as between the two groups (*P* < 0.05). MAP in group B was lower than in group S at 30 min after the start of the operation, 60 min after the start of operation, immediately after drug withdrawal, and immediately after extubation (*P* < 0.05), as shown in Figure 4. No serious anesthetic-related adverse reactions or complications were found in the two groups.

**Table 2:** Comparison of characteristics between bispectral index (B) and standard of care (S) groups.

**Groups**	**B (*n* = 188)**	**S (*n* = 187)**	***Z* value**	***P-*value**
Sex (male/female)	152/36	151/36	—	0.980
Age (years)	1 (1,2)	1 (1,2)	−0.6020	0.547
Weight (kg)	12 (11,13)	12 (10,13)	−1.153	0.249
Surgery type				
Urologic	119	126		
General surgery	29	25		
Oncologic	16	15		
Orthopedic	19	16		
Others	5	5		

*P* > 0.05.

Median (25, 75%).

**Table 3:** Anesthesia time, extubation time, duration of PACU stay, and total propofol administered in the bispectral index (B) and standard of care (S) groups.

**Groups**	**B (*n* = 188)**	**S (*n* = 187)**	***Z* value**	***P*-value**
Anesthesia time (min)	111 (83,141)	111 (82,144)	−0.344	0.731
Extubation time (min)	15 (10,21)	14 (11,20)	−0.068	0.946
Duration in PACU (min)	27 (20,37)	29 (22,39)	−1.285	0.199
Total amount of propofol administered (mg)	288 (223, 391)^a^	264 (217, 335)	−2.035	0.042

^a^*P* < 0.05.

Median (25, 75%).

**Table 4:** BIS values, HR and MAP at each time point in group B and group S ($\bar{x}$±s).

**Time/**	**BIS**		**HR**		**MAP**	
	**B (*n* = 188)**	**S (*n* = 187)**	**B (*n* = 188)**	**S (*n* = 187)**	**B (*n* = 188)**	**S (*n* = 187)**
T1	93 ± 5	94 ± 4	167 ± 21	157 ± 21	81 ± 16	88 ± 11
T2	47 ± 14	45 ± 15	120 ± 26	126 ± 17	66 ± 11	71 ± 14
T3	55 ± 12	56 ± 14	112 ± 17	117 ± 19	65 ± 12	65 ± 10
T4	57 ± 8	61 ± 8	111 ± 13	114 ± 22	61 ± 12	69 ± 11
T5	52 ± 7 ^a^	57 ± 9	102 ± 11	104 ± 15	59 ± 13^a^	71 ± 12
T6	54 ± 7^a^	57 ± 9	97 ± 10	101 ± 13	55 ± 10^a^	70 ± 9
T7	57 ± 6^a^	60 ± 8	92 ± 10	100 ± 17	53 ± 9^a^	72 ± 14
T8	74 ± 4	75 ± 4	113 ± 14	119 ± 14	60 ± 11^a^	76 ± 13

^a^*P* < 0.05, values of BIS, HR and MAP were compared between the group B and the group S at the same time point.

*BIS*, bispectral index; *HR*, hear rate; *MAP*, mean aterial pressure; *T1*, 1 min before induction; *T2*, 1 min after induction (1 min after the induction drugs were administrated); *T3*, immediately after intubation; *T4*, immediately after skin incision; *T5*, 30 min after the start of the operation; *T6*, 60 min after the start of operation; *T7*, immediately after drug withdrawal; *T8*, immediately after extubation.

## Discussion

In this study, after performing a comparison between BIS-guided TIVA anesthesia and according to the variation in the clinical signs guided TIVA anesthesia in younger children, it was found that the use of BIS monitoring in TIVA in younger children during anesthesia cannot reduce the time of extubation and the duration of stay in the PACU.

This study used a manually controlled infusion pump instead of a target-controlled infusion (TCI) pump. Although there are currently some studies on the reliability of target-controlled infusion of propofol in children (19), there are studies showing that there are distinct differences in the time to reach the expected BIS value in different target-controlled infusion modes eye-opening time, and time of extubation (20). In addition, the results showed that there was no significant difference in the administration of propofol between manually-controlled infusion and target-controlled infusion of propofol, indicating that manually-controlled infusion of propofol is a feasible method for children. When propofol infusion was guided by the BIS, no major difference was found between TIVA and TCI (20).

BIS is a value from 0 to 100 calculated based on a certain complex algorithm of adult EEG frequency, not a simple EEG frequency display. BIS technology has undergone a series of upgrades. The four electrode Quattro BIS (Aspect Medical Systems, Norwood, MA, USA) sensor was released in January 2005 and is now recommended for the pediatric population (21). The results of retrospective studies conducted by Carlos et al. demonstrated that using BIS monitoring in adult general anesthesia operations could reduce extubation, time of awakening, duration of stay in the operating room, and PACU. It can also reduce postoperative nausea and vomiting, cognitive dysfunction within 3 months after the operation, postoperative delirium, intraoperative awareness, and adverse events related to anesthetic medications (5). The results of a study conducted by Bhardwaj et al. showed that BIS-guided propofol infusion in children was not superior to the conventional anesthesia method in anesthetic consumption and recovery time (7), and the study conducted by Bresil et al. on continuous infusion of propofol and remifentanil with BIS monitoring in children or adults showed similar results. It was concluded that continuous infusion of propofol and remifentanil with BIS monitoring could not reduce the amount of anesthetic used and the time of extubation in children or adults (22). The results of our study showed that there was no statistically significant difference in the time of extubation and duration of stay in the PACU between the BIS monitoring group and the standard clinical practice group, which was different from that of the adult study by Carlos et al. (5) but consistent with that of a previous study on children by Bhardwaj et al. (16). There was a statistically significant difference in the total amount of propofol administered in our study. The total amount was greater in the BIS group than in the standard clinical practice group. This result was inconsistent with the findings of previous studies on children by Bhardwaj et al. and Bresil et al. (16, 22). The reason may be that the above previous studies were conducted in older children, while our study was aimed at younger children during anesthesia. The anesthesiologist in the standard clinical practice group was relatively cautious to face the younger children when administering the drug. This result was also consistent with those reported in previous literature, showing that there could be a higher risk when manually controlled infusions were conducted using only clinical signs as reference indexes (19).

The results of studies conducted by Tschiedel et al. showed that when the BIS was less than 60, good sedation could be provided during anesthesia in children, intraoperative awareness could be reduced. The depth of anesthesia sedation could be adjusted at any time according to the BIS value to prevent drug overdose (23). In this study, the BIS values of younger children in the BIS group were consistently below 60 during anesthesia at T2–T7, maintaining a good depth of anesthesia, while the BIS values of younger children in group S were all ≥60 at T4–T7. The difference between the two groups was statistically significant. In terms of clinical signs, although there was no statistically significant difference in heart rate between the two groups, the mean arterial pressure in group S was at a high level from half an hour after the start of the operation to immediately after extubation, which was significantly different from that in the BIS group. The results of previous studies in children showed that there was no statistically significant difference in blood pressure, heart rate, and BIS value between the BIS monitoring group and the standard clinical practice group during anesthesia (16, 22). Although one study has shown that the BIS values of children aged 1–11 during anesthesia were higher than 60, that may be due to no muscle relaxant (22). Based on the analysis of the results of the BIS value and mean arterial pressure, it was considered that this was because the anesthesiologist in group S was relatively cautious while administering drugs in younger children during anesthesia. Therefore, there may be a risk of intraoperative awareness if there is shallow anesthesia in group S. These results further explain why the consumption of propofol in group B was larger than that in group S. Nevertheless, there was no difference in the time of extubation between groups B and S, indicating that TIVA with BIS monitoring was superior to the method used in the standard controlled group.

In this study, the mode of continuous infusion of propofol and remifentanil was intraoperatively used. A study has shown that remifentanil can reduce the cardiac index (CI) and increase the plasma concentration of propofol. However, it had no obvious effect on the BIS value (24). Therefore, during the operation, in group B, the anesthesia depth was adjusted by changing the dose of propofol according to the BIS value, while in group S, the depth was adjusted according to the clinical signs.

## Limitations

There were some limitations to this study. First, our BIS machines cannot export continuous data, so we use manual recording of point-in-time data. Although the data displayed by the machine is lagging, all the data are also lagging, so the statistical results are still meaningful. Second, because of younger age, no investigation of intraoperative awareness was performed in the selected children. Therefore, in shallow anesthesia, it can be speculated that there is a possibility of intraoperative awareness. Finally, the single-blind approach may affect the results. These are also the focus of our future research.

## Conclusions

In conclusion, in younger children, TIVA with BIS monitoring cannot reduce extubation time and staying time in the PACU, and also cannot reduce the propofol consumption.

## Data availability statement

The original contributions presented in the study are included in the article/supplementary material, further inquiries can be directed to the corresponding authors.

## Ethics statement

The studies involving human participants were reviewed and approved by Ethical Committee of Beijing Children's Hospital, Capital Medical University, National Center for Children's Health. Written informed consent to participate in this study was provided by the participants' legal guardian/next of kin.

## Author contributions

JZ contributed to the overall study design, data analysis, and revised the paper. FW helped design the study and revised the manuscript. GL helped design the study, performed the experiments, analyzed the data, and wrote the previous versions of the manuscript. LL and XZ performed the experiments and collected the data. All authors have read and approved the final version of this manuscript.

## Conflict of interest

The authors declare that the research was conducted in the absence of any commercial or financial relationships that could be construed as a potential conflict of interest.

The reviewers CW and YS declared a shared parent affiliation with the authors to the handling editor at the time of review.

## Publisher's note

All claims expressed in this article are solely those of the authors and do not necessarily represent those of their affiliated organizations, or those of the publisher, the editors and the reviewers. Any product that may be evaluated in this article, or claim that may be made by its manufacturer, is not guaranteed or endorsed by the publisher.

## Abbreviations

BIS: bispectral index
HR: heart rate
MAP: mean arterial pressure
PACU: post-anesthesia care unit
TIVA: total intravenous anesthesia
ASA: American Society of Anesthesiologist
TCI: target-controlled infusion.
